# Improved Detection of Plasmon Waveguide Resonance Using Diverging Beam, Liquid Crystal Retarder, and Application to Lipid Orientation Determination

**DOI:** 10.3390/s19061402

**Published:** 2019-03-21

**Authors:** Sivan Isaacs, Etienne Harté, Isabel D. Alves, Ibrahim Abdulhalim

**Affiliations:** 1 Department of Electrooptics and Photonics Engineering and The Ilse Katz Institute for Nanoscale Science and Technology, Ben Gurion University of the Negev, Beer Sheva 84105, Israel; 2 CBMN, UMR 5248 CNRS, Université de Bordeaux, Allée Geoffroy St. Hilaire, 33600 Pessac, France; etienne.harte@u-bordeaux.fr (E.H.); i.alves@cbmn.u-bordeaux.fr (I.D.A.)

**Keywords:** surface plasmon resonance, plasmon waveguide resonance, liquid crystal, biosensors

## Abstract

Plasmon waveguide resonance (PWR) sensors exhibit narrow resonances at the two orthogonal polarizations, transverse electric (TE) and transverse magnetic (TM), which are narrower by almost an order of a magnitude than the standard surface plasmon resonance (SPR), and thus the figure of merit is enhanced. This fact is useful for measuring optical anisotropy of materials on the surface and determining the orientation of molecules with high resolution. Using the diverging beam approach and a liquid crystal retarder, we present experimental results by simultaneous detection of TE and TM polarized resonances as well as using fast higher contrast serial detection with a variable liquid crystal retarder. While simultaneous detection makes the system simpler, a serial one has the advantage of obtaining a larger contrast of the resonances and thus an improved signal-to-noise ratio. Although the sensitivity of the PWR resonances is smaller than the standard SPR, the angular width is much smaller, and thus the figure of merit is improved. When the measurement methodology has a high enough angular resolution, as is the one presented here, the PWR becomes advantageous over other SPR modes. The possibility of carrying out exact numerical simulations for anisotropic molecules using the 4 × 4 matrix approach brings another advantage of the PWR over SPR on the possibility of extracting the orientation of molecules adsorbed to the surface. High sensitivity of the TE and TM signals to the anisotropic molecules orientation is found here, and comparison to the experimental data allowed detection of the orientation of lipids on the sensor surface. The molecular orientations cannot be fully determined from the TM polarization alone as in standard SPR, which underlines the additional advantage of the PWR technique.

## 1. Introduction

Surface plasmon resonance (SPR) is a very useful technique for the analysis of small changes in the refractive index (RI), and therefore it is used for chemical and biological sensing and drug development as well as in the study of material sciences [1,2]. Kretschmann–Raether geometry is the most conventional configuration for the excitation of surface plasmon wave (SPW) in several modes, such as angular, spectral, polarimetric, or intensity. The SPR sensor is characterized by several parameters, such as sensitivity, full width at half maximum (FWHM) of the resonance, and penetration depth [3,4,5]. This sensor can be interrogated in three main modes: angular, spectral, and imaging [6]. The traditional SPR sensor has an angular sensitivity of 60–100 (deg/RIU), where RIU stands for refractive index units, penetration depth less than 300 nm, and FWHM of 3 degrees in the angular mode, which is a figure of merit, or FoM, of 20–33 RIU^−1^. Detection limits of SPR sensors vary; however, the best ones reported are based on phase and spectral SPR modes showing detection limits of 10^−7^–10^−8^ RIU. Different structures were suggested to overcome the disadvantages of small penetration depth using infrared light [7] or using long range SPR where a dielectric layer of low RI is added under the metal layer [8,9], and recently, a structure of insulator-metal-insulator (IMI) was designed [10] to obtain ultra-long range penetration depth. Two modes are excited in the IMI structure: one plasmon mode is on the bottom surface of the metal layer, and one plasmon waveguide resonance (PWR) couples surface plasmon and waveguide excitation modes. This PWR mode is obtained with a metal layer and a waveguide layer on top and was first implemented by Salamon et al. for measuring the optical anisotropy of membrane systems [11]. Using rigorous theoretical fitting, the optical guided mode was used to monitor the kinetics of lipid membrane formation [12]. In this structure, the top dielectric layer is used as a waveguide and can sustain guided modes for transverse electric (TE) polarization as well as for transverse magnetic (TM) if it is thick enough. Different works were carried out using this technique for detecting large particles and biological targets [13,14,15,16,17]. The PWR was interrogated mainly in the angular mode [18]. Recently, a study for the spectral mode for both TE and TM polarization was published [19]. A comparison study of the SPR and PWR sensors has been performed showing less sensitivity in the PWR case; however, the FoM was improved because the FWHM was less than that in the SPR case by more than an order of magnitude [20]. When the thickness of the top dielectric layer is small enough so that it cannot support guided modes, the sensor is termed nearly guided wave SPR (NGWSPR), in which the field and sensitivity as well as the figure of merit are enhanced, particularly in the spectral mode [6,21,22,23].

In this paper, we first investigate the coupled waveguide SPR or PWR experimentally using the diverging beam approach [24]. Polarized light at 45° generates the two TE and TM modes simultaneously since the beam contains a wide spectrum of angles though showing weak contrast because at the dip of one polarization (say, TE), the other polarization (TM) will add a background, and vice versa. One way to overcome this problem and improve the contrast is to use a liquid crystal variable retarder (LCVR), which can act as a half wave plate and therefore can switch between the TE and TM modes using a small voltage at high speed. Secondly, we demonstrate the applicability of the PWR sensor for determining the orientation of molecules adsorbed to the surface by analysis of the TE and TM resonance locations and fitting to rigorous theoretical calculations. Due to this fact as well as the enhanced figure of merit from the guided modes that can be made very narrow (<0.1 degree) depending on the waveguide thickness, the PWR sensor can show superiority over the standard SPR approach, if the system is able to resolve the narrow resonances. The combination of the PWR with the simplified high contrast, high signal-to-noise ratio methodology presented here demonstrates the superiority of this technique, which is particularly important for studies of the interaction between molecules and the interaction between molecules and anisotropic thin films such as lipid membranes as well as in materials surface science.

## 2. Methods and Results

**Figure 1:**
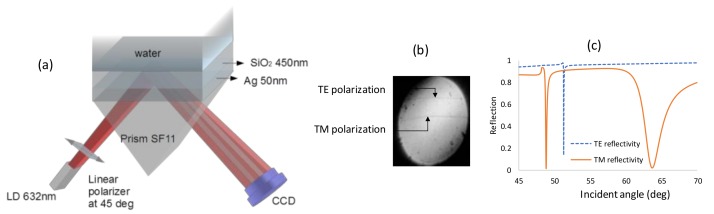
(**a**) Diverging beam setup for coupled plasmon waveguide sensor with the light polarized at 45°. (**b**) Experimental result. (**c**) Simulation results.

Figure 1a shows the setup for the PWR sensor. The experiments were done using the miniature SPR system from Photonicsys Ltd. (www.photonicsys.com). Linearly polarized light at 45° to the incidence plane emerges from a laser diode at a wavelength of 632 nm and is incident at the backside of a 90° prism made of SF11 glass. A SF11 substrate coated with the nominal thicknesses of 50 nm Ag and 450 nm SiO_2_ was placed on top of the prism (coating done at ECI, Evaporated Coatings, Willow Grove, PA, USA). In reality the thickness variations are within 5%. Between the substrate and the prism, a thin layer of index matching oil was introduced. Solutions containing different ratios of water and ethanol were used as the analyte, and the reflected beam was captured with a webcam. Both signals for TE and TM are shown in Figure 1b when the analyte is water; however, the contrast of the two dips is weak because at each of the resonances, 50% of the light does not excite the resonant wave and instead is reflected.

**Figure 2:**
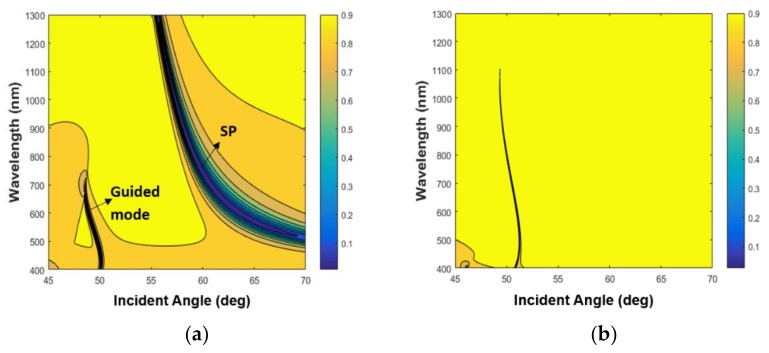
The dispersion relation. (**a**) Tranverse magnetic (TM) mode. (**b**) Transverse electric (TE) mode. The yellow area represents the total internal reflection (TIR) region, and the dark area represents the zero level.

The simulations based on the 2 × 2 Abeles matrix method in Figure 1c confirm that for the TM mode, two dips appear: one is at 63.7°, which is the SPR resonance; and the second is at 48.86°, which is the waveguide mode. The thicknesses that were taken for Ag and SiO_2_ are the nominal ones: 50 nm and 450 nm, respectively. The RIs at 632nm of the prism, Ag, SiO_2_, and water are 1.7877, 0.13612 + i4.01062 [25], 1.4762 [26], and 1.333, respectively. The dispersion relation for this configuration for both TM and TE modes were calculated and shown in Figure 2. In Figure 2a, the dispersion relation for the TM mode shows that the SPR resonance caused by the bottom thick layer of SiO_2_ can be excited on the bottom surface of the metal layer. Since its field distribution is mainly in the SiO_2_ and the metal layer it is not sensitive to the refractive index of the analyte, the sharp TM mode indicates the existence of a guided mode. The surface plasmon (SP) mode is at a higher angle and has a lower contrast, which is why it was not observed with our setup.

**Figure 3:**
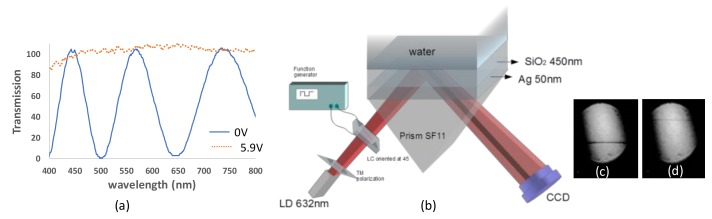
(**a**) The transmission of the LC with the optic axis rotated at 45° with respect to the polarizer axis between crossed polarizers at 0 V and 5.9 V. (**b**) Diverging beam setup incorporating a liquid crystal cell. (**c**) Result for TM polarization. (**d**) Result for TE polarization.

As mentioned, one way to improve the contrast is by using a liquid crystal variable retarder (LCVR). Liquid crystal cells are used as variable retarders with small voltages, allowing the incident light to change its polarization state. To construct the liquid crystal (LC) cell, we used 2 mm glass substrates coated with indium tin oxide (ITO). A thin layer of polymer photoalignment (ROC 108) was deposited on the substrates. After baking them, we exposed the glasses to linearly polarized UV light with an intensity of 0.7 mW for 7 min. The measured cell thickness was 12.53 μm. The cell was filled in vacuum with E44 LC (Merck) by capillary suction. In Figure 3a, the transmission of the LC cell with the optic axis at 45° between cross polarizers is shown both with no voltage and with an applied voltage of 5.9 V. The applied voltage has a square function with a frequency of 1 kHz. At a wavelength of 632 nm, the LC acts as a full waveplate (FWP) at zero voltage while it becomes a half waveplate (HWP) at 5.9 V.

The diverging beam setup with TM polarization and LCVR appears in Figure 3b. Without voltage, the LC keeps the same polarization as the polarizer axis. Based on the measured thickness and the birefringence (Δn = 0.257), the output intensity between crossed polarizers is I = sin^2^(π∆nd/λ), showing a minimum at 632 nm as shown in Figure 3c, but at 5.9 V, the polarization varies, resulting in the TE polarization giving the maximum signal in Figure 3d.

**Figure 4:**
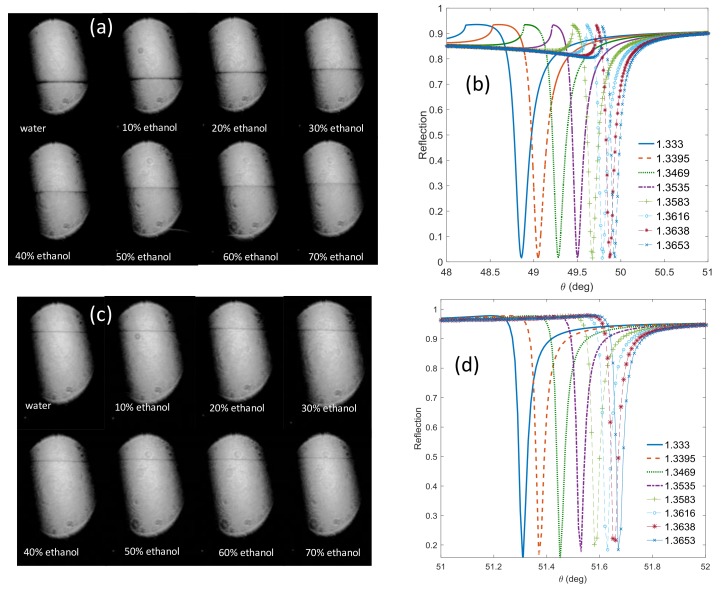
(**a**) TM and (**c**) TE resonance signatures obtained from the camera for different ethanol concentrations in water and the corresponding simulated resonance curves (**b**,**d**), respectively.

The sensitivity study was carried out for different concentrations of water and ethanol ˂70% for both cases of TE and TM polarizations. For TM polarization, the results in Figure 4a show the TM waveguide resonance at different concentrations while Figure 4b shows the corresponding simulated curves. Applying a voltage of 5.9 V to the LC cell changes the polarization state to TE polarization. This case causes excitation of the TE waveguide mode only as it appears in Figure 4c. In Figure 4d, the reflection for the TE mode is simulated.

**Figure 5:**
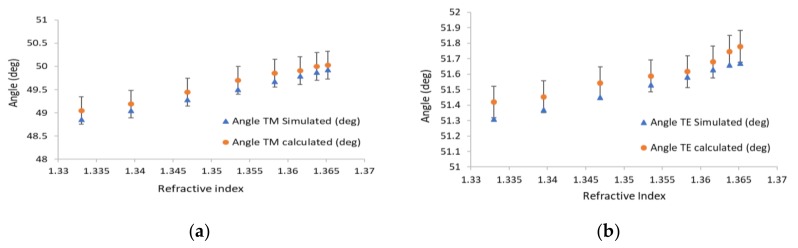
Measured and calculated resonance angles versus refractive index both for (**a**) TM and (**b**) TE; blue triangles indicate the angle of the waveguide resonance, and the circles are the calculated angles.

**Figure 6:**
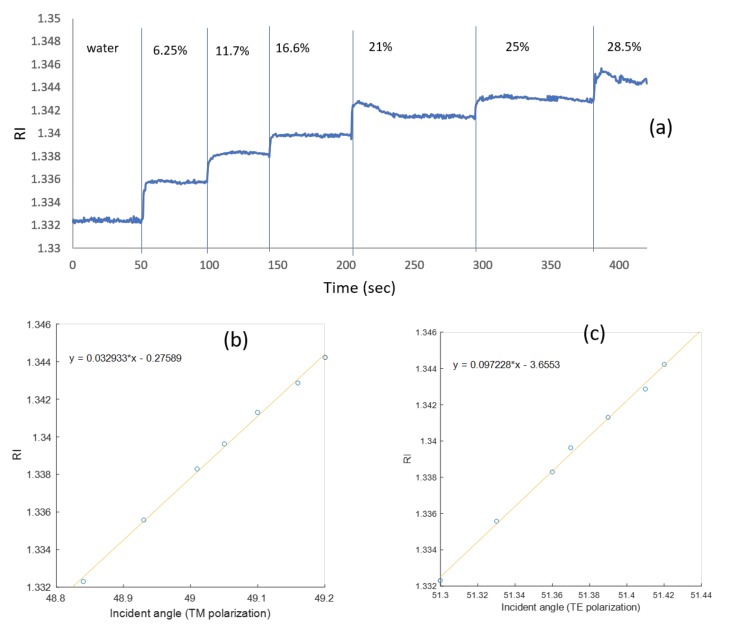
(**a**) Refractive index sensogram for different ethanol water mixtures with TM polarization in serial mode. The relation between the incident angle and the refractive index for (**b**) TM mode and (**c**) TE mode. The angles here are calculated inside the SF11 prism.

In Figure 5a, the blue triangles are the simulated resonance angles for different refractive indices, and the circles are the translated pixel numbers to angles based on Figure 4. From the slope of Figure 5a, the sensitivity of the waveguide resonance for the TM mode is 30 (deg/RIU). The sensitivity for TE polarization is 10 (deg/RIU). In Figure 5b, the blue triangles represent the simulated waveguide angles for different refractive indices, and the black squares are the translated pixel numbers to angles based on Figure 4. The difference between the simulation and the calculation is 0.5%. Note that the widths of the dips for the TE mode are even smaller than the TM case by at least a factor of five. Thanks to the high angular resolution of the Photonicsys system (better than 0.001°), the narrow dips of the PWR structure were easily measured at high speed. A refractive index sensogram for different ethanol and water mixtures is shown in Figure 6a. Knowing the refractive index, we can calculate the incident angles for TE and TM modes as it appears in Figure 6b, c. There is a linear relation between the incident angle and the refractive index for both polarizations, and therefore we have used these as calibration curves. 

## 3. Anisotropic Molecules Orientation Determination

**Figure 7:**
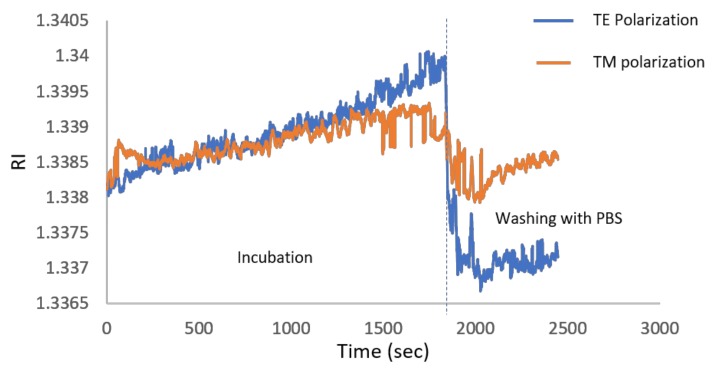
Sensogram during immobilization of lipids to the sensor and then after washing at around 1800 s. The refractive index (RI) difference between the TE and TM signals become more pronounced as the time increases because of the increase in the binding to the surface.

One of the important applications of the PWR sensor (with TM and TE resonances) distinguishing it from standard SPR (with TM only) is to use the polarization dependence for finding molecular orientations of anisotropic molecules on the sensor chip surface. To study this, we incubated the sensor chip for 30 min with a 10 mg/mL suspension of small unilamellar vesicles (SUV) of lipid (phosphatidylcholine, POPC) and then washed them with phosphate buffered saline (PBS). Due to their high membrane curvature, SUVs are known to spontaneously burst upon contact with the hydrophilic outer silica surface of the sensor to form a solid-supported lipid membrane [27]. The formation of such a lipid membrane was monitored using the Photonicsys SPR system. The sensogram in Figure 7 shows the RI after 30 min of incubation at both polarizations. The zero time is the moment of adding the lipid solution. The RI of the buffer alone is 1.335 while the RIs after washing with PBS for TM and TE polarizations are 1.337 and 1.33847, respectively, which is caused by a contribution both from the bilayer and from the buffer in the bulk. Note that these values are lower than the RI at zero time of ~1.3382 because at zero time, the bulk liquid contains both the buffer and the excess lipids (SUVs) that did not fuse with the silica to form the membrane and that were afterwards rinsed. Following simulation and the calibration data shown in Figure 6b,c, the resonance angles for TM and TE polarizations are 58.974° and 51.361°, respectively. The simulation takes into account all the PWR structure layers, the bilayer of 5.5 nm, and the buffer above (with RI of 1.335).

## 4. Discussion

Anisotropic molecules such as lipids and DNA have different refractive indices along their long axis or perpendicular to it. Assuming they are aligned perpendicular to the surface, the refractive index perpendicular to the surface is the extraordinary refractive index (n_e_), which is different from the refractive index parallel to the surface (n_o_). The n_e_ and n_o_ in POPC are 1.526 and 1.4505, respectively [28], and the thickness is approximately 5.5 nm. The setup in Figure 1 can give information on the mass density and distribution. In order to verify, in a more conclusive way, whether a bilayer was formed on the surface, rigorous simulation is likely required. Therefore, the rigorous 4 × 4 matrix approach [29], developed originally for liquid crystals optics, is used, providing the exact solution of light propagation through an anisotropic medium. The generalized field vector of the electromagnetic field tangential components can be defined as $\psi^{T}=\left(E_{x},H_{y},E_{y},-H_{x}\right)$, and its propagation is governed by the following system of first order equations:

(1) $$\frac{d\psi }{dz}=ik_{0}\Delta \psi$$

Here, the Δ matrix is termed the differential propagation matrix, and for propagation in the XZ plane, it is given by:

(2) $$\Delta =\left(\begin{array}{cccc} -\nu_{x}\frac{\varepsilon_{xx}}{\varepsilon_{zz}} & 1-\frac{\nu_{x}{}^{2}}{\varepsilon_{zz}} & -\nu_{x}\frac{\varepsilon_{zx}}{\varepsilon_{zz}} & 0\\ \varepsilon_{xx}-\frac{\varepsilon_{xz}\varepsilon_{zx}}{\varepsilon_{zz}} & -\nu_{x}\frac{\varepsilon_{xz}}{\varepsilon_{zz}} & \varepsilon_{xy}-\frac{\varepsilon_{xz}\varepsilon_{zx}}{\varepsilon_{zz}} & 0\\ 0 & 0 & 0 & 1\\ \varepsilon_{yx}-\frac{\varepsilon_{yz}\varepsilon_{zx}}{\varepsilon_{zz}} & -\nu_{x}\frac{\varepsilon_{yz}}{\varepsilon_{zz}} & \varepsilon_{yy}-\nu_{x}{}^{2}-\frac{\varepsilon_{yz}\varepsilon_{zy}}{\varepsilon_{zz}} & 0 \end{array}\right)$$

**Figure 8:**
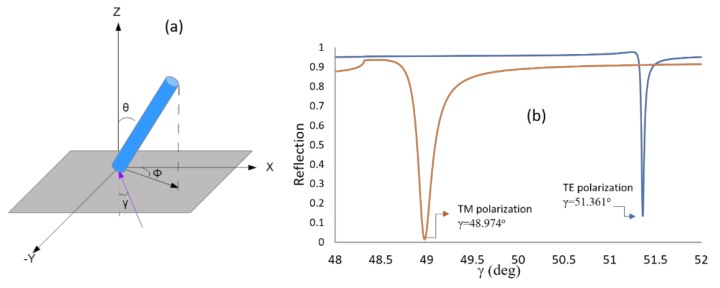
(**a**) Lipid orientation: θ represents the tilt angle, Φ represents the azimuth angle and γ is the incident angle. (**b**) Simulated reflectivity using 4 × 4 matrix approach for TE and TM obtained by changing the tilt and azimuth angles until the dip coincides with the incidence angle values obtained from the experiment. The tilt and azimuth angles obtained are θ = 40.2° and Φ = 15.01°.

Here, *ε_xx_*, *ε_xz_*, *ε_zx_*, *ε_yy_*, *ε_yz_*, and *ε_zy_* are the elements of the dielectric tensor, and γ is the incidence angle inside the prism. For the lipids layer, the angle θ represents the tilt angle (polar), and Φ represents the azimuth angle between the projection of the LC director and the x axis as it appears in Figure 8a. For the expression of the dielectric tensor in terms of the different angles and for more details on the calculation procedure, the readers are referred to Reference [29]. Using this method, we calculated the reflection curve that best fits the experimental result and found the values for the tilt and the azimuth angles to be 40.2° and 15.01°, respectively, as it appears in Figure 8b.

**Figure 9:**
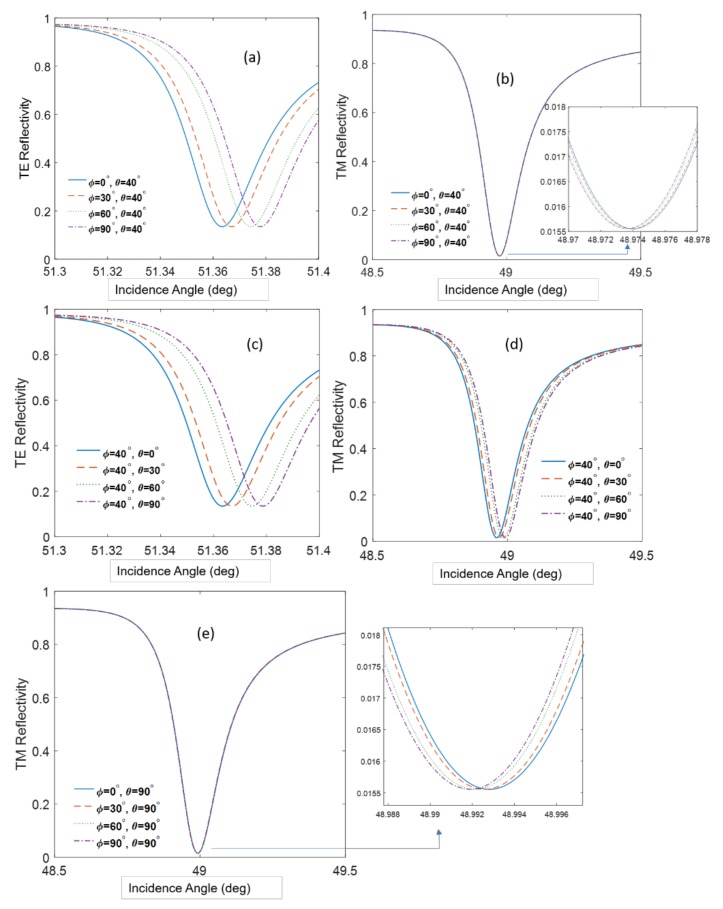
(**a**,**b**) The reflection curves for TE and TM polarization in case θ is constant. (**c**,**d**) The reflection for TE and TM polarization in case Φ is constant. (**e**) TM reflectivity for high tilt angle. The incidence angle corresponds to the internal angle in the SF11 prism.

The numerical solution can also give us the sensitivity dependence on the orientation of the molecule. In case the tilt angle (θ) is constant and the azimuth angle (ϕ) is varying, the reflection was calculated as it appears in Figure 9a,b. The other case where the tilt angle is varying and the azimuth angle is constant (ϕ = 40°) gives the results as shown in Figure 9c,d.

Figure 9 teaches us several important consequences: (i) At a fixed tilt angle, the TE mode resonance angle increases as the azimuth angle increases; (ii) at a fixed tilt angle the, TM mode resonance angle is not sensitive to the azimuth angle, although a tilt angle as large as 90°, the sensitivity slightly increases (Figure 9e); and (iii) at a fixed tilt angle, the TE resonance angle increases while the TM resonance angle decreases. The orientation of anisotropic molecules on surfaces depends on many parameters such as the surface tension of the surface (hydrophilicity), the shape of the molecule, and whether it has special groups or dipoles that interact with the dipoles on the surface [30]. Therefore, it is usually difficult to predict beforehand how a molecule will align itself on a surface, and here we propose a methodology to determine this alignment, because a greater sensitivity of the TE and TM resonances to the tilt and azimuth angle was found. In our case, the orientation angles for the tilt and the azimuth equal approximately 40° and 20°, respectively. Previous reports showed an average tilt angle around 38° [28]; however, so far, there has been no attention to the azimuth angle variation. It is believed that these orientation angles are a function of the molecules–surface interaction and can vary slightly between experiments.

In PWR and SPR experiments with anisotropic molecules, the resonance angle sometimes decreases with the concentration, which is the opposite of our expectations [12,31]. The calculations in Figure 9b show that these unexpected shifts could be due to increasing the azimuth.

Table 1 shows the sensitivity to the azimuth angle orientation for cases in Figure 9b,e where the tilt angle is constant and the azimuth angle is changing. From the table, we conclude that the sensitivity improves slightly for higher tilt angles. Although the changes in the resonance angle are small, they can be observed and sometimes erroneously interpreted as originating from noise or drifts. The PWR allows determination of the polar or azimuth angle orientations, which is contrary to the case of SPR, which cannot give the adequate information from only the TM polarization.

## 5. Potential Applications of the Proposed Methodology

As evidenced above, the PWR sensor and the described technology allows one to determine the molecular orientation of anisotropic oriented objects, as is the case of anisotropic thin films as lipid model membranes. This is a great advantage relative to other plasmon resonance approaches that, due to their exclusive resonances with *p*-pol (TM polarization), lack such information. Determining molecular orientation is fundamental when working with such systems in order to determine if lipids are properly oriented and the membrane model used is pertinent relative to real cellular membranes. As lipid membranes constitute both a selective barrier to the extracellular system and the milieu for accommodating membrane proteins, applications implicating the use of lipid membranes and their investigation by the described technology are certainly quite vast. Two general topics must be mentioned to which classical PWR measurements have substantially contributed: (1) the study of the mechanisms of action and membrane interaction of membrane active peptides (antimicrobial, amyloid, cell penetrating, viral peptides); and (2) the study of the ligand activation and signaling of membrane proteins as G-protein coupled receptors (GPCRs). Regarding point (2), as membrane active peptides share the common point of interacting, perturbing, fusing, translocation through, self-assembling, or other mechanisms implicating the lipid membrane, the characterization of peptide/lipid interactions in terms of affinity, kinetics, and overall mass changes upon peptide action (e.g., mass lost can be an indication of detergent mechanisms or pore formation by the peptides) helps elucidate the peptide mechanism of action (pore formation, lipid membrane disruption, interaction without major lipid organization, formation of domains, etc.). PWR has contributed to the elucidation of the mechanisms of lipid interaction in cell penetration [32,33,34], and for viral [35,36] and amyloid peptides [13,37]. As for studies implicating GPCRs, the possibility of monitoring orientation is quite important because such proteins are quite anisotropic (cylindrical shape) and are totally oriented with their long axis perpendicular to the lipid membrane when in cellular membranes. It is therefore important to know how they are oriented relative to the sensor and the lipid membrane before pursuing further studies. Indeed, PWR was used to follow the reconstitution of GPCRs from a detergent environment to the planar lipid membrane and then to determine protein activity by its capacity to recognize its ligand [38]. Usually, such approaches are carried out by more than one approach and often rely on the use of a high quantity of material and also require labelled materials. PWR has been successful for studies of ligand-activation and receptor conformational changes [39,40,41,42,43], signaling [44,45], lipid impact [35,46], and domain partitioning [47] in GPCR activity, highly contributing to a better understanding the way such receptors function. The proposed methodology in this work provides higher contrast measurement in high speeds and high resolution, yet maintaining simplicity using a single laser diode and camera. It shows clear measurement of the narrowest resonance in TE with FWHM of less than 0.1 degrees, hence the full FoM of around 100 RIU^−1^, which is larger than the standard SPR FoM b ay factor of 3–5 at least.

## 6. Conclusions

To conclude, experimental and theoretical investigations were performed on plasmon waveguide resonance sensor exhibiting both TE and TM resonances using the diverging beam approach. Two methods of obtaining the TE and TM resonances are presented: one simultaneous but with low contrast, and one serial with high contrast using a liquid crystal variable retarder. The sensitivity for TM is 30 (deg/RIU), the sensitivity for TE mode is 10 (deg/RIU), and it remains as a guided wave at the whole RIs range. The resonance dips are an order of magnitude narrower than the standard SPR dip, which is why the figure of merit is larger by a few times. In addition, the dielectric waveguide layer protects the silver layer from any oxidation or degradation, which is another advantage over standard SPR where gold has to be used for its stability even though it is less sensitive than silver. Theoretical simulations using the 2 × 2 Abeles matrix method confirm the experimental findings with isotropic liquid as an analyte while the 4 × 4 matrix approach allowed us to find the orientation of anisotropic molecules adhered to the surface and the sensitivity of the signal to the orientation angles (tilt and azimuth). This study shows that guided mode SPR can be very useful when combined with the diverging beam approach, allowing the study of the orientation of molecules in video rate by detecting the TE and TM simultaneously, or at nearly half the video rate using LCVR but with higher contrast.

## Figures and Tables

**Table 1:** The calculated incidence angle for different azimuth angle.

	(θ = 40°) Incidence Angle (deg)	(θ = 90°) Incidence Angle (deg)
Φ = 0°	48.9741	48.9928
Φ = 30°	48.9739	48.9925
Φ = 60°	48.9737	48.9921
Φ = 90°	48.9736	48.9918
